# No evidence of the role of early chemical exposure in the development of β-cell autoimmunity

**DOI:** 10.1007/s11356-018-3659-6

**Published:** 2018-11-13

**Authors:** Harri M. Salo, Jani Koponen, Hannu Kiviranta, Panu Rantakokko, Jarno Honkanen, Taina Härkönen, Jorma Ilonen, Suvi M. Virtanen, Vallo Tillmann, Mikael Knip, Outi Vaarala, the DIABIMMUNE Study Group

**Affiliations:** 1 Scientific Laboratory, Clinicum, University of Helsinki, Haartmaninkatu 8, 00290 Helsinki, Finland; 2 Department of Health Security, National Institute for Health and Welfare (THL), Kuopio, Finland; 3 Children’s Hospital, University of Helsinki and Helsinki University Hospital, Helsinki, Finland; 4 Research Programs Unit, Diabetes and Obesity, University of Helsinki, Helsinki, Finland; 5 Immunogenetics Laboratory, University of Turku, Turku, Finland; 6 Department of Clinical Microbiology, University of Eastern Finland, Kuopio, Finland; 7 Department of Health, National Institute for Health and Welfare, Helsinki, Finland; 8 School of Health Sciences, University of Tampere, Tampere, Finland; 9 Science Centre, Pirkanmaa Hospital District and Center for Child Health Research, Tampere University and University Hospital, Tampere, Finland; 10 Department of Pediatrics, University of Tartu and Tartu University Hospital, Tartu, Estonia; 11 Folkhälsan Research Center, Helsinki, Finland; 12 Tampere Centre for Child Health Research, Tampere University Hospital, Tampere, Finland

**Keywords:** Human, Chemical exposure, β-cell autoimmunity, Type 1 diabetes, Breastfeeding

## Abstract

Exposure to environmental chemicals can modulate the developing immune system, but its role in the pathogenesis of type 1 diabetes is largely unexplored. Our objective was to study the levels of circulating concentrations of environmental pollutants during the first years of life and their associations with the later risk of diabetes-predictive autoantibodies. From two birth-cohort studies including newborn infants with HLA-conferred susceptibility to type 1 diabetes (FINDIA and DIABIMMUNE), we identified case children with at least one biochemical diabetes-associated autoantibody (*n* = 30–40) and from one to four autoantibody-negative controls per each case child matched for age, gender, diabetes-related HLA-risk, delivery hospital, and, in FINDIA, also dietary intervention group. Plasma levels of 13 persistent organic pollutants and 14 per- and polyfluorinated substances were analyzed in cord blood and plasma samples taken at the age of 12 and 48 months. Both breastfeeding and the geographical living environment showed association with circulating concentrations of some of the chemicals. Breastfeeding-adjusted conditional logistic regression model showed association between decreased plasma HBC concentration at 12-month-old children and the appearance of diabetes-associated autoantibodies (HR, 0.989; 95% Cl, 0.978–1.000; *P* = 0.048). No association was found between the plasma chemical levels and the development of clinical type 1 diabetes. Our results do not support the view that exposure to the studied environmental chemicals during fetal life or early childhood is a significant risk factor for later development of β-cell autoimmunity and type 1 diabetes.

**Electronic supplementary material:**

The online version of this article (10.1007/s11356-018-3659-6) contains supplementary material, which is available to authorized users.

## Introduction

Type 1 diabetes is an autoimmune disease caused by T cell-mediated destruction of the pancreatic β-cells. The disease process takes usually years from its initiation to overt diabetes. In the asymptomatic preclinical period, autoantibodies against several β-cell-derived antigens appear into the peripheral circulation (Bottazzo et al. 1974). These autoantibodies include islet cell antibodies (ICA), insulin autoantibodies (IAA), GAD antibodies (GADA), insulinoma-associated-2 antibodies (IA-2A), and zinc transporter 8 antibodies (ZnT8A) (Pietropaolo et al. 2012). The destruction of the pancreatic β-cells is perceived to be mediated by autoreactive T cells. Overactivation of IFN-γ-secreting Th1 (Foulis et al. 1991; Kallmann et al. 1997) and IL-17-secreting Th17 cells (Honkanen et al. 2010) and impaired function of regulatory T cells (Treg) (Lindley et al. 2005; Putnam et al. 2009) have been reported in animal models of autoimmune diabetes as well as in human type 1 diabetes. In addition, aberrancies in the phenotype and function of dendritic cells have been associated with the development of type 1 diabetes (Nieminen et al. 2012).

In industrialized countries, the widespread use of chemicals has been increasing simultaneously with the rise in the incidence rate of type 1 diabetes (Onkamo et al. 1999). Some of the chemicals are very persistent in the environment, bioaccumulative, and capable of interfering with biological systems at different levels. One of the known harmful effects of environmental pollutants is that they are able to modulate the functions of the human immune system. Exposure of laboratory animals to immunotoxic chemicals at early developmental stages may result in more severe effects on the immune system than exposure during adult life (reviewed in Holladay 1999). Also in humans, exposure in utero and in the first years of life is of special concern since the developing immune system of fetuses and young children is highly vulnerable to toxicant exposure (Holsapple et al. 2004).

Persistent organic pollutants (POPs) are a diverse group of organic compounds including dioxins, polychlorinated biphenyls (PCBs), pesticides, and certain brominated flame-retardants. Exposure to POPs may contribute to the development of autoimmunity, such as systemic lupus erythematosus (Cooper et al. 2008; Holladay 1999). Per- and polyfluorinated substances (PFAS) own attractive water and oil repellent characteristics and have been used in a variety of consumer and industrial applications since the 1950s (Lau et al. 2007). Perfluorooctanoic acid (PFOA) and perfluorooctane sulfonate (PFOS) are the most well-known PFAS. Both have been implicated to have immunotoxic properties (Corsini et al. 2014). Exposure to PFAS has been associated with reduced immune responses to routine childhood immunizations (Grandjean et al. 2012; Granum et al. 2013) and with an increase in the incidence of childhood asthma (Dong et al. 2011). Early life exposure to PFAS occurs both via placental transfer (Apelberg et al. 2007) and breastfeeding (Thomsen et al. 2010).

The possibility that environmental chemicals are involved in the pathogenesis of type 1 diabetes has been debated but has not so far been adequately addressed (Bodin et al. 2015; Howard and Lee 2012). Results from epidemiological studies focusing on a possible link between type 1 diabetes and exposure to POPs are contradictory. Pregnant women with type 1 diabetes had 30% higher levels of serum PCBs than controls (Longnecker and Daniels 2001). In contrast, elevated levels of PCB-153 and dichlorodiphenyldichloroethylene (p,p′-DDE) in maternal serum did not correlate with the development of type 1 diabetes in the offspring in a Swedish cohort (Rignell-Hydbom et al. 2010). In support of PCB effects on autoimmunity, the prevalence of GADA was four times higher in employees in a factory producing PCBs compared to controls (Langer et al. 2002). Several modes of action to trigger or accelerate type 1 diabetes development by chemicals have been implicated. In addition to immunomodulation, chemicals may have direct toxic effects on β-cells, may alter hormone levels, affect the microbiota, or alter intestinal permeability (Bodin et al. 2015).

There is definitely a need for studies analyzing the potential association between early life exposure to environmental chemicals and development of type 1 diabetes. Our hypothesis is that early life exposure to environmental chemicals plays a role in the development and/or progression to type 1 diabetes. In this study, we set out to investigate the association between prenatal and postnatal exposure to environmental chemicals (POPs including PFASs) and the development of β-cell autoimmunity and clinical diabetes.

## Materials and methods

### Study design

The role of prenatal and early life exposure to environmental chemicals in the development of β-cell autoimmunity was studied in children participating in the FINDIA pilot study (the Finnish Dietary Intervention Trial for the Prevention of Type 1 Diabetes) (Vaarala et al. 2012). Exposure to environmental chemicals in young children was studied from plasma samples from 4-year-old participants in the DIABIMMUNE study (Peet et al. 2012).

### Study subjects

In the FINDIA and DIABIMMUNE studies, children with HLA genotypes conferring susceptibility to type 1 diabetes were monitored for the appearance of disease-associated autoantibodies. Cases were selected from children who developed at least one diabetes-associated biochemical autoantibody during the follow-up period (from birth to 6 years of age in FINDIA and from 3 to 5 years of age in DIABIMMUNE). For each case child, from one to four autoantibody-negative control, children matched for age, gender, diabetes-related HLA-risk, delivery hospital, and dietary intervention group (baby milk formula, in FINDIA only) were selected. With these strict matching criteria, we identified 40 cases with plasma samples taken at birth (cord blood), 36 cases with plasma samples taken at 12 months of age, and 30 cases with plasma samples taken at 4 years of age (Table 1). The cord blood samples were obtained between May 2002 and November 2005 and the 12-month samples between May 2003 and November 2006 in three pediatric hospitals in Finland as described earlier (Vaarala et al. 2012). Blood samples from 4-year-old children were drawn between October 2010 and August 2011 in pediatric hospitals of Tartu, Estonia, and Espoo, Finland (12 and 18 case-control pairs, respectively).

**Table 1:** Characteristics of the study subjects

Study	FINDIA		DIABIMMUNE
Origin/age of plasma collection	Cord blood	12 month	48 month
Years of plasma collection	2002–2005	2003–2006	2010–2011
Matched Aab+ cases/Aab− controls	40/111	36/62	30/30
Matched T1D cases^†^/Aab− controls	18/51	16/29	9/7
Seroconversion follow-up	From birth to 6 years		From 3 to 5 years
Age at seroconversion (years)	2.3 (0.5–6.0)		3.1 (3.0–5.0)
Age of T1D diagnosis (years)^†^	7.5 (1.2–12.3)		5.5 (2.6–9.8)
Breastfeeding (months)	9.1 (0.5–26.4)		8.1 (0.5–24.0)

^†^Type 1 diabetes diagnoses were updated by November 2017

The local Ethical Committees approved both the FINDIA and the DIABIMMUNE studies, and the parents gave their written informed consent prior to their child’s participation in these studies.

### Diabetes-associated autoantibodies

In the FINDIA study, blood samples were obtained at the follow-up visits when the children were 3, 6, and 12 months of age and thereafter annually up to the age of 6 years. In the DIABIMMUNE study, blood samples were drawn at 36, 48, and 60 months of age. Plasma samples were collected from fresh heparinized blood samples and stored at − 70 °C until analyzed. Plasma samples from both studies were screened for IAA, GADA, and IA-2A with specific radiobinding assays as previously described (Knip et al. 2010). In addition, DIABIMMUNE samples were also analyzed for ZnT8A (Knip et al. 2010). The cutoff level for autoantibody positivity was 2.80 relative units (RU) for IAA, 5.36 RU for GADA, 0.78 RU for IA-2A, and 0.61 RU for ZnT8A, representing the 99th percentiles in more than 350 Finnish non-diabetic children.

### HLA genotyping

HLA typing of major risk DR-DQ haplotypes for type 1 diabetes was performed with a PCR-based lanthanide-labeled hybridization method using time-resolved fluorometry for detection as described before (Hermann et al. 2003). HLA genotyping in the FINDIA and DIABIMMUNE studies has been described in detail earlier (Peet et al. 2012; Vaarala et al. 2012).

### Chemical analysis

Plasma concentrations of 13 POP compounds including PCBs 118, 138, 153, 156, 170, and 180, pesticides hexachlorobenzene (HCB), β-hexachlorocyclohexane (β-HCH), oxychlordane, *trans*-nonachlor, dichlorodiphenyltrichloroethane (p,p′-DDT), p,p′-DDE, and brominated diphenyl ether BDE-47 and 14 PFAS compounds including perfluorohexanesulfonic acid (PFHxS), perfluoroheptanesulfonic acid (PFHpS), perfluorooctanosulfonic acid (PFOS), perfluorononanosulfonic acid (PFNS), perfluorodecanesulfonic acid (PFDS), perfluorohexanoic acid (PFHxA), perfluoroheptanoic acid (PFHpA), perfluorooctanoic acid (PFOA), perfluorononanoic acid (PFNA), perfluorodecanoic acid (PFDA), perfluoroundecanoic acid (PFUnA), perfluorododecanoic acid (PFDoA), perfluorotridecanoic acid (PFTrA), and perfluorotetradecanoic acid (PFTeA) were analyzed. Details of sample pretreatment, instrumental analysis, and method performance have been described elsewhere (Koponen et al. 2013). In brief, the compounds were extracted from 25 to 200 μL plasma using a two-stage liquid-liquid extraction after spiking with isotope-labeled internal standards. In case of the POP measurements, the case-control pairs were reduced from 39 to 34 at 12 months of age and from 30 to 29 at 48 months of age due to insufficient sample volumes. The POP extract was further purified with a miniaturized silica column. Quantification of the POPs and PFASs was performed by GC-MS/MS and LC-MS/MS, respectively. If the concentration of any chemical was below the limit of quantification (LOQ), then the sample was given the value of the LOQ/2 in the statistical analyses.

### Statistics

Conditional logistic regression was used to estimate the hazard ratios (HR) and 95% confidence intervals (CI) for the association between plasma chemical concentration and the risk of emergence of diabetes-related autoantibodies. Unadjusted analyses and analyses adjusted for the duration of the breastfeeding period were performed. When the concentration of a given chemical was below the LOQ in more than 40% of the samples analyzed, the comparisons between groups were performed using categorized values (below the LOQ vs. above the LOQ). No correction for multiple comparisons was applied; instead, multiplicity issues were taken into account by cautious interpretation of the results. The statistical analyses were done using SPSS 22 package.

## Results

Plasma concentrations of the various chemicals in the FINDIA and DIABIMMUNE children, who did not show any signs of type 1 diabetes progression, i.e., remained negative for diabetes-associated autoantibodies without any type 1 diabetes diagnosis, are presented in Table 2.

**Table 2:** Concentration of plasma chemicals in children without any signs of β-cell autoimmunity and no progression to type 1 diabetes

	Cord blood	12 month	48 month
	*n* = 111	*n* = 62	*n* = 30
HCB, mean (SD), pg/mL	<	100.7 (69.7)	58.6 (29.9)
β-HCH, mean (SD), pg/mL	<	45.4 (35.6)	49.5 (52.5)
Trans-nonachlor, mean (SD), pg/mL	<	24.3 (20.7)	<
p,p′-DDE, mean (SD), pg/mL	41.4 (27.7)	312.1 (276.7)	411.9 (444.9)
PCB-118, mean (SD), pg/mL	<	39.4 (33.5)	42.0 (62.1)
PCB-153, mean (SD), pg/mL	28.3 (103.1)	222.4 (190.2)	132.1 (115.2)
PCB-138, mean (SD), pg/mL	15.5 (10.2)	117.3 (97.9)	79.6 (72.4)
PCB-156, mean (SD), pg/mL	<	13.1 (11.2)	11.5 (11.6)
PCB-180, mean (SD), pg/mL	16.7 (9.8)	99.0 (90.0)	43.4 (37.5)
PCB-170, mean (SD), pg/mL	<	49.8 (43.5)	22.3 (18.5)
BDE-47, mean (SD), pg/mL	<	21.3 (27.4)	<
PFHpA, mean (SD), ng/mL	ND	0.72 (0.77)	<
PFOA, mean (SD), ng/mL	1.77 (1.36)	8.2 (4.7)	2.37 (1.02)
PFNA, mean (SD), ng/mL	ND	1.04 (0.65)	0.52 (0.26)
PFDA, mean (SD), ng/mL	ND	0.37 (0.26)	0.20 (0.13)
PFHxS, mean (SD), ng/mL	ND	0.75 (0.58)	0.30 (0.19)
PFOS, mean (SD), ng/mL	3.6 (1.7)	5.6 (3.1)	1.71 (0.80)

<All samples below detection limit

*ND*, not done

A case: control conditional logistic regression model was built to establish associations between plasma chemical concentrations and the emergence of diabetes-related autoantibodies. Cases and controls were matched for age, gender, HLA risk genotype, and delivery hospital and in the FINDIA study cohort also for the milk formula given to the child.

Unadjusted plasma concentrations of the chemicals analyzed showed no association with the emergence of autoantibodies, HR values being close to 1.0 (Table 3). The only exception was that the group having PFDA above LOQ at 48 months showed an association with the appearance of autoantibodies (HR, 4.1; 95% Cl, 1.1–14.3; *P* = 0.028). The PFDA concentrations were below the detection limit in 46% of the 48-month samples, and therefore, the PFDA results were analyzed as categorized variables, below the LOQ or above the LOQ.

**Table 3:** Unadjusted odds ratios and odds ratios adjusted for the duration of breastfeeding (BF) for the appearance of diabetes-associated autoantibodies

	Cord blood	12 month		48 month	
	Unadjusted	Unadjusted	BF adjusted	Unadjusted	BF adjusted
HCB	0.86 (0.52–1.43)‡	0.99 (0.99–1.00)	0.99* (0.98–1.00)	1.01 (0.99–1.03)	1.03 (0.99–1.08)
β-HCH	0.94 (0.80–1.09)‡	0.99 (0.98–1.00)	0.99 (0.98–1.00)	1.00 (0.99–1.01)	0.99 (0.98–1.01)
Trans-nonachlor	1.06 (0.86–1.29)‡	1.00 (0.98–1.02)	0.99 (0.97–1.02)	1.00 (0.95–1.05)	0.99 (0.91–1.08)
p,p′-DDE	1.01 (1.00–1.02)	1.00 (1.00–1.00)	1.00 (1.00–1.00)	1.00 (1.00–1.00)	1.00 (1.00–1.00)
PCB-118	1.07 (0.90–1.28)‡	1.00 (0.99–1.00)	1.00 (0.98–1.01)	1.00 (0.99–1.01)	0.99 (0.97–1.01)
PCB-153	1.01 (0.99–1.03)	1.00 (1.00–1.00)	1.00 (1.00–1.00)	1.00 (1.00–1.00)	1.00 (0.99–1.01)
PCB-138	1.02 (0.98–1.05)	1.00 (0.99–1.00)	1.00 (0.99–1.00)	1.00 (1.00–1.01)	1.00 (0.99–1.01)
PCB-156	†	0.99 (0.95–1.03)	0.99 (0.94–1.03)	1.00 (0.96–1.04)	0.96 (0.89–1.04)
PCB-180	1.02 (0.98–1.05)	1.00 (0.99–1.00)	1.00 (0.99–1.01)	1.00 (0.99–1.01)	0.99 (0.98–1.01)
PCB-170	1.00 (0.87–1.14)‡	1.00 (0.99–1.01)	1.00 (0.98–1.01)	1.00 (0.98–1.03)	0.99 (0.95–1.03)
BDE-47	1.00 (0.97–1.03)‡	0.99 (0.97–1.01)	0.99 (0.97–1.01)	1.05 (0.96–1.14)	1.08 (0.96–1.21)
PFHpA	ND	1.39 (0.78–2.50)	1.55 (0.74–3.21)	19.1 (0.01–25032)‡	6.89 (0.02–1784)‡
PFOA	0.90 (0.67–1.22)	1.01 (0.93–1.10)	1.03 (0.92–1.15)	1.16 (0.80–1.67)	1.09 (0.85–1.41)
PFNA	ND	1.07 (0.56–2.05)	1.39 (0.61–3.17)	4.20 (0.53–33.3)	2.24 (0.09–55.9)
PFDA	ND	1.58 (0.31–7.97)	5.59 (0.61–51.4)	4.10* (1.09–14.3)‡	3.78 (0.76–18.8)‡
PFHxS	ND	0.76 (0.34–1.73)	0.88 (0.37–2.12)	5.50 (0.19–154)‡	3.90 (0.01–1396)‡
PFOS	1.08 (0.96–1.22)	1.00 (0.88–1.14)	1.07 (0.88–1.30)	1.31 (0.78–2.19)	1.29 (0.33–5.03)

Hazard ratios (95% CL) presented

*ND*, not done

*P < 0.05

†All samples below detection limit, not analyzed

‡Analyzed as categorized to above LOQ vs. below LOQ

The duration of the breastfeeding period based on maternal questionnaires was available for 92.5% of children in the FINDIA study and for 84.9% of children in the DIABIMMUNE study. Breastfeeding alone did not associate with the appearance of autoantibodies in either study cohort. As expected, the duration of the breastfeeding period correlated directly with the plasma chemical concentrations (Supplemental Table S1). We performed conditional logistic regression analysis adjusted for the duration of the breastfeeding period (Table 3). Breastfeeding period was categorized into four groups, namely, 0–4, 4–8, 8–12, and more than 12 months of breastfeeding. The only significant finding in the conditional logistic regression analyses adjusted for the duration of breastfeeding was the observation that low plasma HCB concentrations in 12-month-old children were associated with the appearance of diabetes-associated autoantibodies (HR, 0.989; 95% Cl, 0.978–1.000; *P* = 0.048).

For both study cohorts, the type 1 diabetes diagnoses were updated as of November 2017 (Table 1). Almost all children with type 1 diabetes were derived from the group of autoantibody positive cases, and accordingly, it was possible to establish a matched type 1 diabetes case: control set for conditional logistic regression as well. The follow-up for type 1 diabetes was much longer than the follow-up for autoantibodies, and some children who were initially classified as autoantibody negative controls were later diagnosed with type 1 diabetes. These children were excluded from the control groups and included in the type 1 diabetes case group. When defining children diagnosed with overt type 1 diabetes as a case, no associations with plasma chemical concentrations were observed. That was the true both for unadjusted and adjusted analyses.

### Matching factors

Among the matching factors, only the HLA risk genotype did not associate with plasma chemical concentrations.

#### Gender

Cord blood plasma PFOS concentrations were higher in boys (4.2 ± 3.3 ng/mL; mean ± SD) than those in girls (3.1 ± 1.6 ng/mL; *P* = 0.001, Mann-Whitney *U* test). No other gender-related differences were observed.

= 0.028. The PFDA concentrations were below the detection limit in 46% of the 48-month samples, and therefore, the PFDA results were analyzed as categorized variables, below the LOQ or above the LOQ

#### Geographic location

Geographic location affected the circulating concentrations of environmental chemicals. Statistically significant findings are presented for FINDIA in Table 4 and for DIABIMMUNE in Table 5. In the FINDIA study, three Finnish regions were compared. The infants residing in the capital region of Helsinki had higher concentrations of p,p′-DDE, PCB-153, PCB-138, PFOA, and PFOS in cord blood plasma than children residing in smaller cities (Jyväskylä and Kuopio; Table 4) or their surroundings. The same phenomenon was observed for the plasma concentrations of PFNA, PFDA, and PFHxS in 12-month-old infants (Table 4).

**Table 4:** Plasma concentrations of environmental chemicals in cord blood and 12-month-old children by geographical location

Chemical	Helsinki	Jyväskylä	Kuopio	*P value
Cord blood	*n* = 30	*n* = 92	*n* = 79	
p,p′-DDE, mean (SD), pg/mL	66.8 (54.7)	42.6 (24.5)	39.0 (30.0)	0.005
PCB-153, mean (SD), pg/mL	40.7 (29.8)	29.8 (14.3)	26.7 (17.6)	0.004
PCB-138, mean (SD), pg/mL	23.6 (16.4)	17.5 (7.8)	15.9 (9.3)	0.002
12-month-old children	*n* = 31	*n* = 52	*n* = 48	
PFOA, mean (SD), ng/mL	2.2 (2.0)	1.3 (0.7)	1.8 (1.2)	0.002
PFOS, mean (SD), ng/mL	4.2 (2.1)	3.7 (1.9)	3.5 (3.5)	0.030
PFNA, mean (SD), ng/mL	1.36 (0.70)	1.02 (0.62)	0.87 (0.54)	0.009
PFDA, mean (SD), ng/mL	0.46 (0.23)	0.38 (0.24)	0.32 (0.25)	0.003
PFHxS, mean (SD), ng/mL	0.89 (0.40)	0.80 (0.60)	0.54 (0.51)	0.001

^*^P value from the Kruskal-Wallis test; only statistically significant results are presented

**Table 5:** Plasma concentrations of environmental chemicals in 48-month-old children by geographical location

Chemical	Espoo (*n* = 30)	Tartu (*n* = 92)	*P value
HCB, mean (SD), pg/mL	40.7 (15.5)	73.8 (36.8)	< 0.001
β-HCH, mean (SD), pg/mL	17.8 (6.7)	71.6 (54.6)	< 0.001
p,p′-DDE, mean (SD), pg/mL	134.8 (104.0)	675.9 (676.6)	< 0.001
PCB-118, mean (SD), pg/mL	15.3 (10.0)	54.1 (61.0)	< 0.001
PCB-138, mean (SD), pg/mL	52.8 (36.6)	99.4 (86.5)	0.04
PFOA, mean (SD), ng/mL	3.08 (0.84)	2.49 (3.81)	< 0.001

^*^P value from the Mann-Whitney *U* test. Only statistically significant results are presented

In the DIABIMMUNE study, the 48-month-old children living in the Estonian city of Tartu and its adjacent areas had higher plasma levels of HCB, β-HCH, p,p′-DDE, PCB-118, and PCB-138 than children living in the city of Espoo, Finland (Table 5). In contrast, children residing in Espoo had higher concentrations of plasma PFOA than children residing in Tartu.

#### Milk formula group

In the FINDIA study, the participants were randomized to be weaned to three different milk formulas. One of the milk formulas was offered to each participant, and breastfeeding was encouraged. A fourth study group comprised children who used no milk formula and relied solely on breastfeeding as his/her milk intake. Plasma chemical concentrations did not differ between the three milk formula groups at the age of 12 months. However, solely breastfed children had higher concentrations of eight POP and five PFAS compounds when combined with pooled milk formula groups (Supplemental Table S2).

## Conclusions

In our study, we analyzed the circulating concentrations of a multitude of environmental pollutants in two matched case-control series. We could not observe any definite associations between increased exposure to chemical pollutants at birth, at 12 or at 48 months of age, and risk of β-cell autoimmunity. The current work indicates that prenatal or early childhood exposure to POPs, including PFASs, is not an apparent risk factor for later β-cell autoimmunity. To our knowledge, this is the first report based on HLA-matched case-control series where exposure to environmental pollutants and the development of β-cell autoimmunity and type 1 diabetes have been studied.

In 48-month-old children, PFDA was above the LOQ in 34% of the autoantibody-negative children, in 63% of the autoantibody-positive children, and in 88% of the children diagnosed with type 1 diabetes. PFDA has been demonstrated to interfere with the function of thyroid hormones in in vitro studies (Long et al. 2013), and endocrine disruption is an interesting mode of action of PFDA in biological systems. It has been shown that PFDA, at concentrations of 100 ng/mL and above, can impair LPS-induced release of TNF-α in peripheral blood leukocytes and prevent LPS-induced I-κB degradation (Corsini et al. 2012). However, it should be mentioned that since PFDA was present at very low concentrations (less than 1 ng/mL) in the current study, the results do not really support any association between elevated circulating concentrations of PFDA and β-cell autoimmunity and/or emergence of type 1 diabetes.

Plasma HCB concentrations at the age of 12 months were actually decreased in case children who developed diabetes-associated autoantibodies by the age of 6 years when compared to children who remained autoantibody-negative, but only after adjustment for breastfeeding. It is of interest that an inverse association has been reported earlier between HCB and circulating concentrations of IFN-γ, indicating that exposure to HCB may downregulate Th1 immunity (Daniel et al. 2001), which has been implicated to be involved in immune-mediated β-cell destruction. Although the present study could be interpreted as indicating slower progression to β-cell destruction in children with increased HCB concentrations, which is to some extent supported by existing literature, there are also arguments against such a view. First, the decrease in plasma levels of HCB in affected children was present only in plasma samples taken at 12 months of age. No changes in plasma levels of HCB were seen in samples taken at birth or at 48 months of age. Second, this finding should be interpreted cautiously since the comparisons were not corrected for multiple comparisons. Third, the higher circulating concentrations of chemicals in children without any signs of β-cell autoimmunity may be explained by other factors protecting against diabetes, e.g., their nutritional pattern. It is known that long-chain fatty acids from fish, the main source of POPs, may protect from β-cell autoimmunity (Rignell-Hydbom et al. 2010). As a conclusion, without further investigations, it is too early to make the statement that increased exposure to HCB protects from β-cell autoimmunity or from type 1 diabetes. Merely, the current work indicates that exposure to HCB, at current concentrations, is not harmful in relation to the development of type 1 diabetes.

The present study was designed to investigate whether early exposure to environmental chemicals associates with β-cell destruction. Although the number of cases, especially for type 1 diabetes cases, is rather low, the value of the present study lays in powerful matching of pairs that were included in the statistical analysis. The confounding factors introduced by age, gender, geographical location, and milk formula groups could be eliminated and further, the effect of the duration of the breastfeeding period could be adjusted for in conditional logistic regression analyses.

We found higher PFOS concentrations in cord blood plasma in boys than in girls, which is in line with earlier studies (Wang et al. 2011). The circulating concentrations of several POPs were higher in Estonian children than those in Finnish children. These are mainly chemicals used in the industry and agriculture before their global restrictions, the usage history of which may well explain the current findings. However, the incidence of type 1 diabetes is substantially lower in Estonia than in the less exposed Finnish population (Harjutsalo et al. 2013; Teeaar et al. 2010) suggesting that exposure to these POPs is not involved in the disease process resulting in type 1 diabetes.

The current study is in line with earlier observations that breastfeeding is an important route of certain persistent chemicals (Kiviranta et al. 1999). Although the current study does not support the protective role of breastfeeding, it should be mentioned that some surveys have shown that breastfeeding may protect against type 1 diabetes (Pereira et al. 2014).

The duration of breastfeeding and geographical location were found to be associated with plasma concentrations of several persistent organic pollutants and per- and polyfluorinated substances in early life. This is in line with previous studies. Taken together, our results suggest that exposure to persistent organic pollutants and per- and polyfluorinated substances, at current levels, does not have any effect on the induction of β-cell autoimmunity or progression from β-cell autoimmunity to clinical type 1 diabetes.

## Electronic supplementary material

ESM 1 (DOCX 16 kb)
