# Supplementary material for: No evidence of the role of early chemical exposure in the development of β-cell autoimmunity
Source: Environ Sci Pollut Res Int. 2018 Nov 13;26(2):1370–8. doi: 10.1007/s11356-018-3659-6 (PMC6331740; doi:10.1007/s11356-018-3659-6)
Supplement: Supplementary file 1 — (DOCX 16 kb) [file 11356_2018_3659_MOESM1_ESM.docx]

SUPPLEMENTARY DATA

This appendix has been provided by the authors to give readers additional information about their work.

Supplement to: “**No Evidence of the Role for Early Chemical Exposure in the Development of β-CellAutoimmunity”** by Harri M. Salo, Jani Koponen, Hannu Kiviranta, Panu Rantakokko, Jarno Honkanen, Taina Härkönen, Jorma Ilonen, Suvi M. Virtanen, Vallo Tillmann, Mikael Knip, Outi Vaarala, and the DIABIMMUNE Study Group

**Table S1 – Correlation between breast feeding period and plasma concentrations of environmental chemicals.**

|  | 12 month | 48 month |
| --- | --- | --- |
| HCB | .655^*^ | .526^*^ |
| bHCH | .508^*^ | .309^*^ |
| tNONaclor | .534^*^ | .499^*^ |
| DDE | .455^*^ | .323^*^ |
| PCB118 | .591^*^ | .352^*^ |
| PCB153 | .598^*^ | .516^*^ |
| PCB138 | .605^*^ | .488^*^ |
| PCB156 | .537^*^ | .431^*^ |
| PCB180 | .600^*^ | .542^*^ |
| PCB170 | .589^*^ | .549^*^ |
| PFHpA | .304^*^ |  |
| PFOA | .453^*^ | .201 |
| PFNA | .470^*^ | .312^*^ |
| PFDA | .379^*^ | .238 |
| PFUnA | .326^*^ | .214 |
| PFHxS | .341^*^ | .365^*^ |
| PFHpS | .413^*^ |  |
| PFOS | .592^*^ | .404^*^ |

Pearsson correlation R-values presented when R>0.2

*. Correlation is significant at the 0.05 level (2-tailed).

**Table S2 – Association of breastfeeding with the plasma concentrations of persistent organic pollutants in 12-month-old children**

| Chemical | Breastfed (n=35) | Formula (n=101) | **P* |
| --- | --- | --- | --- |
| HCB, mean (SD), pg/mL | 115.3 (58.4) | 86.2 (62.7) | 0.004 |
| Transnonachlor, mean (SD), pg/mL | 29.4 (17.0) | 23.2 (17.5) | 0.004 |
| PCB118, mean (SD), pg/mL | 46.1 (26.1) | 37.0 (32.8) | 0.01 |
| PCB153, mean (SD), pg/mL | 271.6 (154.2) | 196.1 (173.8) | 0.003 |
| PCB138, mean (SD), pg/mL | 141.5 (79.3) | 106.3 (91.1) | 0.005 |
| PCB156, mean (SD), pg/mL | 16.4 (10.4) | 12.2 (8.7) | 0.002 |
| PCB180, mean (SD), pg/mL | 126.8 (84.2) | 83.1 (74.7) | 0.001 |
| PCB170, mean (SD), pg/mL | 64.0 (41.2) | 41.1 (35.1) | 0.001 |
| PFOA, mean (SD), ng/mL | 9.8 (4.7) | 7.6 (4.3) | 0.006 |
| PFNA, mean (SD), ng/mL | 1.3 (0.6) | 1.0 (0.6) | 0.01 |
| PFDA, mean (SD), ng/mL | 0.5 (0.3) | 0.4 (0.2) | 0.01 |
| PFHxS, mean (SD), ng/mL | 1.0 (0.7) | 0.6 (0.5) | <0.001 |
| PFOS, mean (SD), ng/mL | 6.9 (3.1) | 4.9 (2.8) | <0.001 |

^*^*P* value from the Mann-Whitney test. Only significant associations are shown.
